# Epstein–Barr Virus Serology Associated With Persistent Oral Human Papillomavirus Infections in Men

**DOI:** 10.1111/jop.70015

**Published:** 2025-08-07

**Authors:** Sanni Rinne, Birgitta Michels, Julia Butt, Kari Syrjänen, Seija Grenman, Tim Waterboer, Stina Syrjänen, Karolina Louvanto

**Affiliations:** ^1^ Department of Obstetrics and Gynecology, Faculty of Medicine and Health Technology Tampere University Tampere Finland; ^2^ German Cancer Research Center (DKFZ) Heidelberg Germany; ^3^ SMW Consultants Ltd. Kaarina Finland; ^4^ Department of Obstetrics and Gynecology Turku University Hospital, University of Turku Turku Finland; ^5^ Department of Oral Pathology, Institute of Dentistry, Faculty of Medicine University of Turku Turku Finland; ^6^ Department of Pathology Turku University Hospital Turku Finland; ^7^ Department of Obstetrics and Gynecology Tampere University Hospital Tampere Finland

**Keywords:** EBV antibodies, EBV serology, Epstein–Barr virus (EBV), human papillomavirus (HPV), oral HPV infections

## Abstract

**Background:**

Most people acquire Epstein–Barr virus (EBV) and certain human papillomaviruses (HPVs) during their lifetime. HPV‐related oropharyngeal carcinomas have increased in recent decades, particularly among men. The role of coinfection with viruses like EBV on HPV outcomes is unclear. We investigated potential associations between EBV serology and longitudinal outcomes of oral HPV infections in men.

**Methods:**

This study included 119 men from the Finnish Family HPV Study who were followed up for 3 years. Blood and oral cavity samples were collected at baseline, 12‐, 24‐, and 36‐month follow‐up visits. HPV was genotyped with the Multimetrix assay, and the serum IgG antibodies of EBV proteins Zebra, EA‐D, EBNA, and VCAp18 were measured with fluorescent bead‐based multiplex serology. Univariate regression analysis was used to measure the strength of the association between different variables.

**Results:**

Most participants (99.2%; *n* = 118) were EBV‐seropositive with stable antibody titers throughout the follow‐up. Self‐reported history of atopy was positively associated with elevated EBNA‐1 levels, with OR 7.43 (95% CI: 1.39–39.76). EBV seropositivity with high titers and elevated EA‐D levels alone increased the risk of type‐specific oral HPV persistence for Types 16, 18, 33, and 51, with OR 4.20 (95% CI: 1.09–16.19) and OR 6.23 (95% CI: 1.19–32.75), respectively.

**Conclusions:**

Most of the participants were EBV‐seropositive as expected. Elevated EA‐D antibody levels and being EBV‐seropositive with high titers significantly increased the risk of type‐specific oral HPV persistence among these men.

## 1 Introduction

Epstein–Barr virus (EBV) was the first human oncogenic virus identified 60 years ago. EBV DNA has been found in different anatomical sites and various malignancies [1, 2], with the strongest causal associations in the development of Hodgkin's lymphoma, Burkitt's lymphoma, nasopharyngeal carcinoma, and gastric carcinoma [3]. EBV, also known as Human herpes virus 4 (HHV4), belongs to the Herpesviridae family. Like other herpesviruses, EBV is known to remain latent in the human body after an acute infection and may reactivate later in life.

EBV is a highly prevalent virus, with an estimated seroprevalence of > 90% in the adult population [4]. EBV is known to cause a clinical infection, infectious mononucleosis, but asymptomatic infections are also common [4]. The primary transmission route is through saliva and oral secretions, deep kissing being the most important predisposing factor for acquiring the virus in adolescence and adulthood [4, 5]. Other potential factors promoting EBV transmission are linked to various sexual activities [6, 7, 8]. After the primary infection, the virus establishes latency in B cells and can sporadically reactivate, releasing viral DNA into saliva and blood. Reactivation from latency to the lytic cycle may occur under specific conditions, often driven by factors that disrupt immune surveillance, such as stress or immune suppression [9, 10].

EBV IgG antibodies in the serum reveal a past EBV infection, while IgM serology is used to diagnose a primary infection [11, 12]. The IgG antibodies of some EBV antigens have been studied for their oncogenic properties, including the antigens Zebra (BZLF1), EA‐D (early antigen‐diffuse), EBNA‐1 (EBV nuclear antigen 1) and VCAp18 (viral capsid antigen p18). The role of these antibodies has been studied recently in gastric and nasopharyngeal cancers, and consistently elevated antibody titers could be associated with EBV‐related oncogenesis [4, 13, 14, 15].

A subgroup of alpha‐human papillomaviruses (HPVs) comprises oncogenic types, best known for their causal role in carcinomas of the anogenital tract, especially in the cervix. These mucosal high‐risk (HR) alpha papillomaviruses are also implicated in the etiology of a subgroup of head and neck cancers (HNSCCs), particularly, those arising in the oropharynx [16]. Globally, the prevalence of oral HPV infections among men ranges from 4.4% to 10.1%, with higher prevalences seen typically in Europe as well as Northern and Southern America [17, 18, 19, 20]. Correspondingly, the incidence of HNSCCs has steadily increased over recent decades, especially among men [21, 22]; however, the underlying drivers differ by region. In developed countries, the rise is more strongly associated with HPV‐related cases, whereas in developing countries, traditional risk factors such as tobacco use remain predominant [21]. Regional trends also vary: declines have been reported in France, Spain, Brazil, and Hong Kong (range of −4% to −27%), while increases have been observed in the United Kingdom, Australia, Japan, and the United States (range of +3.7% to +21.3%) [21].

Previous studies on oral squamous cell carcinoma (OSCC), a subtype of HNSCC, have disclosed some connections between EBV‐ and HPV‐related malignancies, but the results have been inconclusive [23, 24]. Recently, the presence of EBV DNA in the oral cavity (oral gargle samples) has been shown to impact the persistence of oral HPV16 infections among men [25]. At present, the possible relationships between EBV and HPV have been incompletely studied, albeit these viruses can infect epithelial cells at the same anatomical sites, including the oral cavity and oropharynx. In cases of viral coinfection, pathogens may interact with each other or indirectly through the host's resources or immune system, and therefore, may contribute to transmission dynamics, clinical progression, and management of the infection, potentially playing a critical role in cancer progression [26, 27]. The present study was designed (1) to examine whether elevated EBV antibody levels might be associated with oral HPV infection outcomes among healthy young men and (2) the potential risk factors of high EBV‐antibody titers.

## 2 Material and Methods

### 2.1 The Finnish Family HPV (FFHPV) Study

This study included 131 men from the FFHPV Study that has been conducted jointly at the Department of Oral Pathology, Turku University, and the Department of Obstetrics and Gynecology, Turku University Hospital since 1998 until the present. The study was originally designed to explore the dynamics and the natural history of HPV infections among the members (mother, father, child) of Finnish families [28, 29]. The FFHPV cohort included 329 mothers, 131 fathers, and their 331 offspring followed up for 6 years. The original study protocol and its amendments have been approved by the Research Ethics Committee of Turku University Hospital, Finland (#3/1998, #2/2006, 45/180/2010, TO7/008/2014, and 329/2018). Informed written consent was obtained from all participants of the study.

### 2.2 Sample Collection and Questionnaire

Oral scrapings from the buccal mucosa were collected with a brush (Cytobrush, MedScand, Malmö, Sweden), and blood samples for serological analyses were taken at baseline and at 12‐, 24‐, 36‐, and 74‐month follow‐up visits as described earlier [29, 30]. At baseline, all participants responded to a structured questionnaire concerning their demographics as well as various sexual practices.

### 2.3 HPV Genotyping

The HPV DNA genotyping in the oral scrapings was performed with a multiplex HPV genotyping kit with modifications (Multimetrix; Progen Biotechnik GmbH) as previously described [31]. In total, 24 low‐risk (LR; HPV 6, 11, 42, 43, 44 and 70) and HR (HPV 16, 18, 26, 31, 33, 35, 39, 45, 51, 52, 53, 56, 58, 59, 66, 68, 73 and 82) HPV genotypes were identified.

### 2.4 EBV Serology

Serum IgG antibodies to four EBV proteins: Zebra, EA‐D, EBNA, and VCAp18, were analyzed with fluorescent bead‐based multiplex serology at the German Cancer Research Center (DKFZ) (Heidelberg, Germany) as previously described [32]. The MFI cut‐off values for seropositivity were previously determined by DKFZ for Zebra, EA‐D, EBNA, and VCAp18 antibodies, as 176, 367, 1500, and 1802, respectively. The subject was classified as EBV‐seropositive if at least two out of the four antibodies exceeded these cut‐off values.

### 2.5 Outcomes of EBV Serology in Men

The final study cohort was 119 men who had at least two or more EBV IgG serology data available during the follow‐up. Of these men: *n* = 84 had EBV serology data available from all four follow‐up visits, 21 from three, and 14 men from two visits. EBV serology outcomes during the 36‐month follow‐up period were grouped into: (1) always seropositive (all samples were classified as seropositive), (2) always seronegative (all samples were seronegative), and (3) seroconverted (an initial negative sample, followed by positive sample). No cases of antibody decay or fluctuating antibody levels were observed.

### 2.6 Statistical Analyses

In statistical analyses, the different EBV antigens (Zebra, EA‐D, EBV, and VCAp18) were evaluated separately and as different antigen combinations. Correlations between individual antigens at each time point were analyzed by Spearman rank correlation coefficient, stratified as low (0.00–0.40), moderate (0.40–0.60), and high (0.60–1.00). The strength of the association between different covariates and HPV infection outcomes was measured with unconditional logistic regression and with a multivariate model adjusted for the participants' age, number of sexual partners, and history of atopy. In these analyses, the MFI values of individual EBV antibodies were divided into tertiles (low, middle, and high antibody levels). All statistical analyses were performed using Stata 16.1 (STATA Corp., TX), all *p*‐values being two‐sided and *p* < 0.05 being considered statistically significant.

## 3 Results

Practically all men, 99.2% (*n* = 118), were seropositive throughout the 36‐month follow‐up (Table 1). No completely seronegative men were observed, but one had a seroconversion to all four antigens during the follow‐up. The highest mean antibody titers were detected against the VCAp18 antigen, and nearly all participants (98.3%) were seropositive to this antigen throughout the follow‐up period. As determinants of the EBV‐seropositivity, the most frequent antigen combination was EBNA and VCAp18, 90.8% (*n* = 108) of the study subjects being seropositive to this combination, while 80.7% (*n* = 96) of the participants were seropositive to all four EBV antigens throughout the follow‐up period.

**TABLE 1:** Outcomes of seroreactivity to individual EBV antigens (Zebra, EA‐D, EBNA‐1, and VCAp18) during the 36‐month follow‐up.

EBV serology outcomes		*n* (%)	MFI mean	SD	Min	Max
Always EBV‐seropositive^a^		118 (99.2)				
	Zebra	Cut‐off 176	4872.3	3060.3	7	13 385
	EA‐D	Cut‐off 367	4436.8	3131.8	89	15 735
	EBNA‐1	Cut‐off 1500	7983.6	3268.0	16	17 283
	VCAp18	Cut‐off 1802	12323.7	3164.4	364	23 056
Antigen‐specific outcomes^b^						
Always positive by antigen						
	Zebra	109 (91.6)	5293.5	2831.8	306	13 385
	EA‐D	113 (95.0)	4621.3	3080.3	385	15 735
	EBNA‐1	109 (91.6)	8439.8	2788.5	1718	17 283
	VCAp18	117 (98.3)	12413.1	3013.6	6114	23 056
All four antigens are positive simultaneously		96 (80.7)				
	Zebra		5299.7	2831.9	397	13 385
	EA‐D		5001.8	3119.2	412	15 735
	EBNA‐1		8591.9	2801.9	1718	17 283
	VCAp18		12605.8	3096.4	6404	23 056
Zebra, EBNA‐1, and VCAp18 positive simultaneously		99 (83.2)				
	Zebra		5241.6	2806.6	397	13 385
	EBNA‐1		8493.8	2823.5	1718	17 283
	VCAp18		12485.5	3054.6	6114	23 056
EA‐D, EBNA‐1, VCAp18 positive simultaneously		104 (87.4)				
	EA‐D		4738.3	3133.6	385	15 735
	EBNA‐1		8520.6	2784.8	1718	17 283
	VCAp18		12516.3	3062.8	6114	23 056
EBNA‐1, VCAp18 positive simultaneously		108 (90.8)				
	EBNA‐1		8432.3	2794.5	1718	17 283
	VCAp18		12473.7	3044.7	6114	23 056
Zebra, EA‐D positive simultaneously		105 (88.2)				
	Zebra		5372.0	2852.5	306	13 385
	EA‐D		4852.9	3073.2	412	15 735

^a^ EBV seropositivity was defined by positive co‐testing to two or more EBV proteins: Zebra, EA‐D, EBNA‐1, and VCAp18.

^b^ Seropositivity to different combinations of EBV antigens.

**FIGURE 1:**
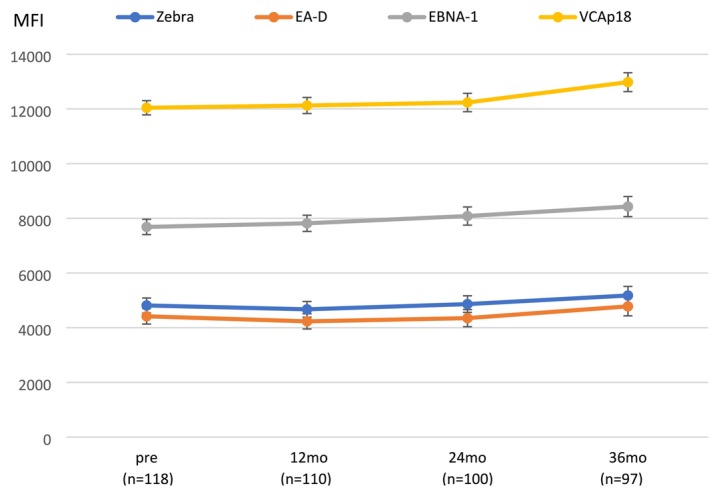
Mean MFI levels of EBV IgG‐antibodies to Zebra, EA‐D, EBNA‐1, and VCAp18 antigens at each visit throughout the 36‐month follow‐up period. Error bars represent 95% confidence intervals (95% CI).

The mean MFI values of the EBV antibody levels among seropositive men during the 36‐month follow‐up are summarized in Figure 1. The levels of all antibody titers remained remarkably stable throughout the follow‐up, with a slight (non‐significant) increase in all titers toward the end of the follow‐up period of 36 months.

The bivariate correlations between the four EBV antibodies are given in (Table 2). The correlations between Zebra and EA‐D antibody levels were moderate at 12‐ and 36‐month follow‐up visits, with *R* of 0.40 and 0.41 (*p* < 0.001), respectively. Interestingly, there was only one significant correlation between EBNA and VCAp18 titers at the 24‐month visit, *R* of 0.41 (*p* < 0.001). All remaining correlations between different antibody titers were insignificant.

**TABLE 2:** Bivariate correlations (Spearman's rho) of the antibody levels between the four EBV antigens (Zebra, EA‐D, EBNA‐1, and VCAp18) at each follow‐up.

	EBNA‐1	Zebra	EA‐D	VCAp18
Baseline				
EBNA‐1	1.00			
Zebra	0.02	1.00		
EA‐D	0.27*	0.30**	1.00	
VCAp18	0.34**	0.14	0.23*	1.00
12‐months				
EBNA‐1	1.00			
Zebra	0.08	1.00		
EA‐D	0.24*	0.40**	1.00	
VCAp18	0.37**	0.14	0.25*	1.00
24‐months				
EBNA‐1	1.00			
Zebra	0.04	1.00		
EA‐D	0.27*	0.39**	1.00	
VCAp18	0.41**	0.15	0.34**	1.00
36‐months				
EBNA‐1	1.00			
Zebra	0.09	1.00		
EA‐D	0.25*	0.41**	1.00	
VCAp18	0.38**	0.18	0.34**	1.00

* *p* < 0.05.

** *p* < 0.001.

A wide variety of health‐ and sexual behavior‐related variables were evaluated for their associations with EBV antibody levels (as tertiles) (Table 3). After adjustment with participants' age, number of sexual partners history of atopy significantly increased the likelihood of elevated EBNA‐1 levels, with OR 7.43 (95% CI: 1.23–46.70, *p* = 0.019). In addition, with the adjusted model practice of anal sex increased the likelihood of elevated VCAp18 levels with OR 2.92 (95% CI: 1.06–8.00, *p* = 0.037). All other tested associations were statistically nonsignificant.

**TABLE 3:** Factors associated with high EBV antibody levels^a^ to Zebra, EA‐D, EBNA‐1, and VCAp18 over a 36‐month follow‐up period, adjusted for age, number of sexual partners, and history of atopy.

		Zebra	EA–D	EBNA–1	VCAp18
			OR (95% CI)		
Number of sexual partners (*n* = 112)	0–2	1.0	1.0	1.0	1.0
	3–5	0.79 (0.20–3.21)	0.77 (0.19–3.12)	0.56 (0.14–2.21)	0.49 (0.12–1.99)
	6–10	1.19 (0.31–4.57)	1.34 (0.35–5.14)	0.87 (0.14–2.21)	1.26 (0.34–4.64)
	≥ 10	1.11 (0.31–3.91)	1.07 (0.30–3.78)	0.34 (0.09–1.25)	0.70 (0.20–2.41)
Number of sexual partners before the age of 20 (*n* = 111)	0–2	1.0	1.0	1.0	1.0
	3–5	0.86 (0.32–2.31)	1.36 (0.52–3.54)	0.97 (0.35–2.72)	1.76 (0.65–4.73)
	5–10	1.06 (0.27–4.18)	0.35 (0.08–1.52)	0.76 (0.16–3.56)	1.00 (0.23–4.30)
	≥ 10	0.59 (0.11–3.30)	0.23 (0.03–1.50)	0.59 (0.09–4.08)	1.55 (0.29–8.33)
First sexual intercourse above 16 (*n* = 112)	Yes	1.29 (0.55–3.01)	1.16 (0.50–2.67)	0.60 (0.24–1.48)	0.71 (0.30–1.64)
Practice of oral sex (*n* = 111)	Never	1.0	1.0	1.0	1.0
	Sometimes	0.45 (0.13–1.58)	0.82 (0.23–2.92)	0.83 (0.22–3.12)	0.77 (0.22–2.74)
	Regularly	0.75 (0.18–3.08)	0.77 (0.18–3.27)	1.48 (0.34–6.51)	1.16 (0.28–4.80)
Reported use of a condom (*n* = 37)	Never	1.0	1.0	1.0	1.0
	Sometimes	0.57 (0.03–12.22)	0.27 (0.01–6.19)	0.22 (0.04–1.36)	0.61 (0.10–3.56)
	Always	0.27 (0.01–8.18)	1.55 (0.05–44.33)	N/C	N/C
Anal sex (*n* = 111)	Yes	1.36 (0.50–3.70)	1.27 (0.50–3.45)	1.01 (0.35–2.95)	**2.92 (1.06–8.00)** *
Other reported sexually transmitted diseases (*n* = 36)^b^	Yes	1.85 (0.13–25.76)	N/C	7.85 (0.46–134.97)	N/C
Allergies (*n* = 109)	Yes	2.03 (0.88–4.70)	1.02 (0.44–2.34)	1.26 (0.53–3.00)	1.78 (0.77–4.10)
Atopy (*n* = 109)	Yes	0.58 (0.11–3.06)	1.04 (0.23–4.68)	**7.43 (1.39–39.76)** **	0.59 (0.11–3.07)

*Note*: Statistically significant values in bold.

^a^ Highest MFI tertial of the antibody levels.

^b^ Other sexually transmitted diseases include chlamydia and genital herpes.

* *p* = 0.037.

** *p* = 0.019.

Different outcomes of oral HPV, including incident oral HPV (*n* = 47), oral type‐specific HPV persistence (*n* = 19), long‐term (> 24‐month) persistent oral HPV16 infections (*n* = 7), and oral HPV infection clearance (*n* = 38) were used as endpoints in estimating the potential impact of EBV serology (Table 4). The duration of type‐specific persistence ranged from 4 to 48 months with a median of 24 months, and the group included HPV genotypes 16, 6, 18, 33, 51, and additionally multiple infections with HPV16. Of the individual antigens, the highest tertile of EA‐D antibodies had a significant association with the type‐specific persistent oral HPV infection group, with Types 16, 18, 33, and 51, OR 6.23 (95% CI: 1.19–32.75, *p* = 0.031). Being classified as EBV‐seropositive (throughout the follow‐up) with high titers of antibodies to at least two antigens also increased the likelihood of type‐specific persistent oral HPV infections, with Types 16, 18, and 33, OR 4.20 (95% CI 1.09–16.19, *p* = 0.037). No other significant associations were disclosed between EBV serology and oral HPV infections. The distribution of high and mid/low EA‐D antibody levels among participants with persistent type‐specific oral HPV infections is shown in Figure S1. Figure S2 presents the distribution of different HPV genotypes, with HPV16 being the most frequently detected and most infections persisting for over 24 months. Notably, all participants with persistent HPV6 infections had low to mid‐level EA‐D antibody levels, whereas persistent infections with genotypes 18, 33, and 51 were observed exclusively in participants with high EA‐D antibody levels.

**TABLE 4:** The associations of EBV Zebra, EA‐D, EBNA‐1, and VCAp18 antibodies to the outcomes of oral HPV infections during the 36‐month follow‐up.

EBV antigen	MFI–tertials	Long–term persistent oral HPV16 infection^b^ (*n* = 7)	Incident oral HPV infection (*n* = 47)	Type–specific persistence of HPV infection (*n* = 19)	Clearance of oral HPV infection (*n* = 38)
Zebra	low	1.00	1.00	1.00	1.00
	mid	0.22 (0.01–3.98)	1.16 (0.43–3.12)	0.74 (0.19–2.90)	0.89 (0.31–2.53)
	high	0.29 (0.03–2.69)	1.32 (0.47–3.70)	1.33 (0.36–4.92)	1.14 (0.39–3.35)
EA–D	low	1.00	1.00	1.00	1.00
	mid	0.50 (0.03–7.99)	1.14 (0.42–3.09)	2.66 (0.45–15.69)	0.69 (0.25–1.91)
	high	2.00 (0.22–17.89)	1.30 (0.48–3.51)	**6.23 (1.19–32.75)** *	0.53 (0.18–1.59)
EBNA–1	low	1.00	1.00	1.00	1.00
	mid	1.50 (0.15–15.46)	0.51 (0.18–1.42)	0.57 (0.15–2.12)	0.52 (0.18–1.54)
	high	4.50 (0.41–49.6)	0.59 (0.21–1.61)	0.48 (0.12–1.86)	0.52 (0.18–1.54)
VCAp18	low	1.00	1.00	1.00	1.00
	mid	0.89 (0.06–12.25)	0.63 (0.23–1.72)	0.83 (0.22–3.13)	0.69 (0.24–2.05)
	high	8.00 (0.58–110.26)	1.06 (0.40–2.86)	N/C	1.17 (0.41–3.34)
**Antigen combinations**					
Simultaneous seropositivity to EBV antigens	≥ 2^a^	2.00 (0.21–18.69)	1.55 (0.45–5.29)	**4.20 (1.09–16.19)** **	0.24 (0.03–2.15)
	all 4	1.25 (0.16–9.54)	0.79 (0.26–2.39)	0.85 (0.20–3.74)	0.60 (0.19–1.84)
	Zebra, EBNA–1, VCAp18	0.83 (0.10–6.78)	1.37 (0.40–4.66)	1.37 (0.26–7.29)	0.85 (0.26–2.80)
	EBNA–1, VCAp18, EA–D	3.00 (0.36–34.20)	0.43 (0.10–1.85)	0.53 (0.08–3.49)	0.33 (0.08–1.44)
	EBNA–1, VCAp18	2.00 (0.17–24.07)	1.43 (0.23–9.00)	1.13 (0.11–11.66)	0.73 (0.14–3.87)
	Zebra, EA–D	0.55 (0.03–10.37)	0.93 (0.28–3.12)	1.14 (0.21–6.26)	0.89 (0.25–3.17)

*Note*: Statistically significant values in bold.

^a^ Co–testing seropositive to 2 or more of the 4 antigens in the highest antibody tertial at every timepoint.

^b^ Defined as > 24–month persistence; Univariate logistic regression analysis was used to estimate ORs; MFI levels of antigen tertials: Zebra (low = 176–4185, mid = 4186–7682, high = 7683–13385), EA–D (low = 367–3490, mid = 3491–6333, high = 6334–15735), EBNA–1 (low = 1500–8432, mid = 8433–11120, high = 11121–17283), VCAp18 (low = 1802–13673, mid = 13674–16415, high = 16416–23056).

* *p* = 0.031.

** *p* = 0.037.

## 4 Discussion

We assessed the EBV IgG serology and its association with oral HPV infection outcomes among 118 men from the FFHPV Study. In this cohort, EBV‐seropositivity was common, at 99.2% of all participants, and the antibody levels remained stable throughout the follow‐up period. Interestingly, a self‐reported history of atopy was significantly associated with elevated EBNA‐1 antigen levels. In addition, high titers of EA‐D antigen, as well as being EBV‐seropositive (i.e., cotesting positive for at least two of the four antibodies) with high titers, were associated with a significantly increased risk for type‐specific persistent oral HPV infection with types 16, 18, 33, and 51.

EBV is a widely spread oncogenic virus, with an estimated population prevalence exceeding 90% among adults [4]. Thus, the seroprevalence of 99% among young male participants in our cohort is in line with these estimates. Furthermore, the high antibody levels of our participants remained stable throughout the follow‐up period, as expected [4]. Of the four EBV antigens, seropositivity to VCAp18 antigen was the most prevalent, with 98.3% of participants testing constantly positive. This finding is in agreement with the previously presented estimates that most, if not all, people develop viral capsid antigen (VCA) IgG antibodies after initial EBV exposure, and the antibody levels remain stable for the rest of a person's life [5, 13]. Seroprevalence to Zebra, EBNA‐1, and EA‐D antigens was 91.6%, 91.6%, and 95.0%, respectively. Also, these data corroborate the previous reports on seropositivity to these antigens, leveling off at 80–100% among healthy adults [13]. Zebra acts as a transcriptional activator that disrupts chromatin structure, inducing transition from latency to lytic replication, while EA‐D promotes viral DNA replication and may interact with host DNA repair machinery [14, 33, 34]. EBNA‐1, a latent cycle protein, is essential for maintenance of the EBV episome in latent cells but may also modulate host immunity and oncogenic signaling pathways during reactivation [34, 35].

The primary transmission site of EBV is through saliva, but sexual activity has also been discussed as a potential source of increased seroprevalence of EBV [7, 8]. The present results failed to disclose any evidence to support this connection, because none of the factors linked to sexual behavior, for example, the number of sexual partners, were associated with elevated EBV antibody levels. Interestingly, a self‐reported history of atopy increased the risk of elevated EBNA‐1 levels. Indeed, EBV‐seropositivity and its association with atopy and allergic diseases have been studied previously, but with conflicting results. Accordingly, some studies failed to disclose any associations between EBV and increased risk of early atopy [36]. In contrast, some others report EBV as a protective factor against atopy and allergic diseases, and others state that EBV may be a risk factor for acquiring atopic diseases [37, 38]. Our finding suggests that there might be an association between atopy and EBNA‐1 antibody levels, but unfortunately, no detailed data are available on EBV exposure and onset of atopy to draw more firm conclusions on this association.

Some interesting observations were made on the possible connection between EBV serology and oral HPV infection outcomes. Elevated levels of EA‐D antibodies as well as testing constantly EBV‐seropositive (by the predefined criteria) with high titers throughout the follow‐up both increased the risk of persistent type‐specific oral HPV infections. This study is the first to demonstrate such an association between EBV serology and outcomes of oral HPV infection. High titers of EA‐D antibodies might indicate reactivation of EBV infection, which could potentially modulate local immune responses or epithelial integrity, thereby influencing HPV persistence. Given that persistent oral HPV infection is a key risk factor for oropharyngeal squamous cell carcinoma, especially HPV16, our findings could support the hypothesis that EBV may act as a cofactor in viral oncogenesis, like its established role in nasopharyngeal and gastric cancers. In line with this, Dickey et al. reported that oral EBV DNA has an impact on persisting oral HPV16 infections [25]. In our study, oral mucosa was not sampled for EBV DNA, limiting direct conclusions about active coinfection. Future studies combining serology and mucosal EBV DNA detection are warranted to better characterize coinfection dynamics. Additionally, it is noteworthy to address the small sample size of our study, with only 17 men having a persistent oral HPV infection. Nonetheless, the combination of serological and DNA‐based evidence suggests that the association of EBV and oral HPV infections would be worth additional studies, particularly because the natural history of oral HPV infection and the predictors of its persistence remain fairly unknown [22].

We also assessed the bivariate correlations between the antibody levels of the four EBV antigens, disclosing the strongest correlation between EBNA‐1 and VCAp18. Of utmost interest are the recent observations reporting that a high seroreactivity to these two antigens was associated with an increased risk of gastric cancer [13]. In addition, high titers to these two EBV antibodies simultaneously also indicate past EBV infection or its reactivation. In the present study, however, we could not find any association between these EBV antibody titers and oral HPV infection outcomes.

One of the main strengths of our study is the longitudinal setting, with a prospective follow‐up of 36 months. To the best of our knowledge, no long‐term follow‐up studies have examined EBV serology and its associations with oral HPV infection outcomes in men. In addition, most of the previous studies on EBV and HPV have focused on DNA from established tumors, in contrast to the association between EBV serology and the natural history of HPV infections as assessed in our study. The potential limitations of our study include progressive loss of participants toward the end of follow‐up, potential reporting bias in the questionnaires, limited sample size regarding oral HPV infections, and most importantly, the missing data on oral EBV DNA as considering only the EBV serology of our participants may not be an ideal study setting for testing our study hypothesis. From a clinical perspective, identifying EBV‐seropositive individuals, especially with high EA‐D titers, could aid in risk stratification for persistent oral HPV infections, paving the way for targeted surveillance or preventive strategies, especially in HR male populations. Future studies incorporating mucosal EBV DNA detection and clinical endpoints are needed to assess the utility of serology in guiding such strategies.

## 5 Conclusions

Almost all men in our study were EBV seropositive throughout the follow‐up period, and a reported history of atopy was associated with elevated EBV antibody levels. Importantly, testing EBV‐seropositive and presenting with high‐level EA‐D antibodies significantly increased the risk of persistent type‐specific oral HPV infection, particularly for HR types such as HPV16, 18, and 33. These findings highlight a potential role for EBV in modulating the persistence of oral HPV, a key event in HPV‐related oropharyngeal carcinogenesis. Future research should integrate mucosal EBV detection, evaluate clinical risk models, and explore the potential utility of EBV serology in identifying individuals at increased risk for persistent oral HPV infections and related malignancies.

## Author Contributions

**Sanni Rinne:** formal analysis, investigation, visualization, writing – original draft, writing – review and editing. **Birgitta Michels:** data curation, formal analysis, investigation, methodology, software. **Julia Butt:** data curation, formal analysis, investigation, methodology, software, writing – review and editing. **Kari Syrjänen:** data curation, formal analysis, investigation, software, validation, writing – review and editing. **Seija Grenman:** conceptualization, data curation, investigation, methodology, writing – review and editing. **Tim Waterboer:** data curation, formal analysis, investigation, methodology, software, validation, writing – review and editing. **Stina Syrjänen:** conceptualization, data curation, formal analysis, funding acquisition, investigation, methodology, project administration, resources, software, supervision, validation, visualization, writing – review and editing. **Karolina Louvanto:** conceptualization, data curation, formal analysis, funding acquisition, investigation, methodology, project administration, resources, software, supervision, validation, visualization, writing – review and editing.

## Conflicts of Interest

T.W. serves on advisory boards for Merck (MSD) Sharp & Dohme. The other authors declare no conflicts of interest.

## Peer Review

The peer review history for this article is available at https://www.webofscience.com/api/gateway/wos/peer‐review/10.1111/jop.70015.

## Supporting information


**Figure S1:** Mean EA‐D antibody levels among participants with persistent type‐specific oral HPV infections. Participants were categorized based on EA‐D antibody levels: those in the highest tertile were classified as having high antibody levels, while those in the lower two tertiles were grouped as low/mid‐level. Error bars indicate 95% confidence intervals for the mean antibody levels.

**Figure S2:** Number of type‐specific persistent oral HPV infections by genotype, duration of persistence, and EA‐D antibody level. Persistence duration was categorized into three groups < 12, 12–24, and > 24 months. The HPV genotypes detected included types 6, 16, 18, 33, and 51.

## Data Availability

The data generated in this study are available upon request.
