# Supplementary material for: Epstein–Barr Virus Serology Associated With Persistent Oral Human Papillomavirus Infections in Men
Source: J Oral Pathol Med. 2025 Aug 7;54(8):733–41. doi: 10.1111/jop.70015 (PMC12419979; doi:10.1111/jop.70015)
Supplement: Supplementary file 1 — Figure S1: Mean EA‐D antibody levels among participants with persistent type‐specific oral HPV infections. Participants were categorized based on EA‐D antibody levels: those in the highest tertile were classified as having high antibody levels, while those in the lower two tertiles were grouped as low/mid‐level. Error bars indicate 95% confidence intervals for the mean antibody levels. [file JOP-54-733-s002.docx]

**Supplementary Figure 1.** Mean EA-D antibody levels among participants with persistent type-specific oral HPV infections. Participants were categorized based on EA-D antibody levels: those in the highest tertile were classified as having high antibody levels, while those in the lower two tertiles were grouped as low/mid-level. Error bars indicate 95% confidence intervals for the mean antibody levels.

MFI
