# Supplementary material for: Epstein–Barr Virus Serology Associated With Persistent Oral Human Papillomavirus Infections in Men
Source: J Oral Pathol Med. 2025 Aug 7;54(8):733–41. doi: 10.1111/jop.70015 (PMC12419979; doi:10.1111/jop.70015)
Supplement: Supplementary file 2 — Figure S2: Number of type‐specific persistent oral HPV infections by genotype, duration of persistence, and EA‐D antibody level. Persistence duration was categorized into three groups < 12, 12–24, and > 24 months. The HPV genotypes detected included types 6, 16, 18, 33, and 51. [file JOP-54-733-s001.docx]

**Supplementary Figure 2.** Number of type-specific persistent oral HPV infections by genotype, duration of persistence and EA-D antibody level. Persistence duration was categorized into three groups <12 months, 12-24 months, and >24 months. The HPV genotypes detected included types 6, 16, 18, 33, and 51.
